# Advancements in Understanding the Hide-and-Seek Strategy of Hibernating Breast Cancer Cells and Their Implications in Oncology from a Broader Perspective: A Comprehensive Overview

**DOI:** 10.3390/cimb46080492

**Published:** 2024-08-01

**Authors:** Aiman Al-Ruwishan, Bushra Amer, Ahmed Salem, Ahmed Abdi, Namoonga Chimpandu, Abdelmonem Esa, Alexandros Melemenis, Muhammad Zubair Saleem, Roselit Mathew, Yaser Gamallat

**Affiliations:** 1 Space for Research Initiative, Research Horizons, London NW10 2PU, UK; 2 Department of Family Medicine, College of Human Medicine, Michigan State University, East Lansing, MI 48824, USA; 3 Department of Biological and Biochemical Sciences, Faculty of Chemical Technology, University of Pardubice, 53210 Pardubice, Czech Republic; 4 Independent Researcher, Uxbridge UB9 6JH, UKnchimpandu@yahoo.com (N.C.);; 5 Department of Pharmacology and Systems Physiology, College of Medicine, University of Cincinnati, Cincinnati, OH 45221, USA; saleemmr@ucmail.uc.edu; 6 Department of Oncology, Biochemistry and Molecular Biology, and Laboratory Medicine, University of Calgary, Calgary, AB T2N 1N4, Canada

**Keywords:** breast cancer dormancy, molecular targeting, G9a, metastatic relapse, therapeutic resistance, biomolecular pathways

## Abstract

Despite recent advancements in technology, breast cancer still poses a significant threat, often resulting in fatal consequences. While early detection and treatments have shown some promise, many breast cancer patients continue to struggle with the persistent fear of the disease returning. This fear is valid, as breast cancer cells can lay dormant for years before remerging, evading traditional treatments like a game of hide and seek. The biology of these dormant breast cancer cells presents a crucial yet poorly understood challenge in clinical settings. In this review, we aim to explore the mysterious world of dormant breast cancer cells and their significant impact on patient outcomes and prognosis. We shed light on the elusive role of the G9a enzyme and many other epigenetic factors in breast cancer recurrence, highlighting its potential as a target for eliminating dormant cancer cells and preventing disease relapse. Through this comprehensive review, we not only emphasise the urgency of unravelling the dynamics of dormant breast cancer cells to improve patient outcomes and advance personalised oncology but also provide a guide for fellow researchers. By clearly outlining the clinical and research gaps surrounding dormant breast cancer cells from a molecular perspective, we aim to inspire further exploration of this critical area, ultimately leading to improved patient care and treatment strategies.

## 1. Introduction

Breast cancer remains a leading cause of cancer-related mortality worldwide [1], primarily due to disease recurrence and metastasis [2]. Despite significant advancements in early detection and treatment modalities in the past 57 years, a subset of breast cancer cells evade therapy-induced cell death and enter a state of dormancy, leading to disease relapse years or even decades after initial diagnosis and treatment [3,4,5]. The phenomenon of cancer cell dormancy [6], particularly in breast cancer, has garnered increasing attention in recent years due to its clinical significance and therapeutic implications [7,8]. In this review, we aim to provide a comprehensive overview of hibernating breast cancer cells, elucidating the underlying mechanisms governing their dormancy, reactivation, and therapeutic resistance. By dissecting the complex interplay between cancer cells and their microenvironments, we seek to identify novel targets and therapeutic strategies to eradicate dormant cancer cells and prevent disease recurrence.

## 2. Dormancy in Breast Cancer

It is important to define the term “tumour dormancy” before discussing the different known mechanisms that contribute to the development of hibernating breast cancer cells. Tumour dormancy is a clinically recognised phenomenon; it refers to a protracted quiescent state in which tumour cells exist but disease progression is not observable. Breast cancer is particularly well known for protracted asymptomatic periods of no disease evidence, lasting up to 25 years, followed by a return [9,10].

Therefore, tumour dormancy is a long-lasting quiescent state in which tumour cells are present but disease progression is not yet observable in patients [11]. Tumour dormancy has also been documented in renal, prostate, and thyroid malignancies, as well as melanoma and B-cell lymphoma, but late recurrences are uncommon in lung and colon cancers [12,13].

A number of experimental models to address the phenomenon of tumour dormancy have been developed based on current data; dormant cancer cells appear to be able to survive either by mitotic arrest, which is a complete withdrawal from the cell cycle, or by slow, cell death-balanced proliferation [14,15].

**Figure 1:**
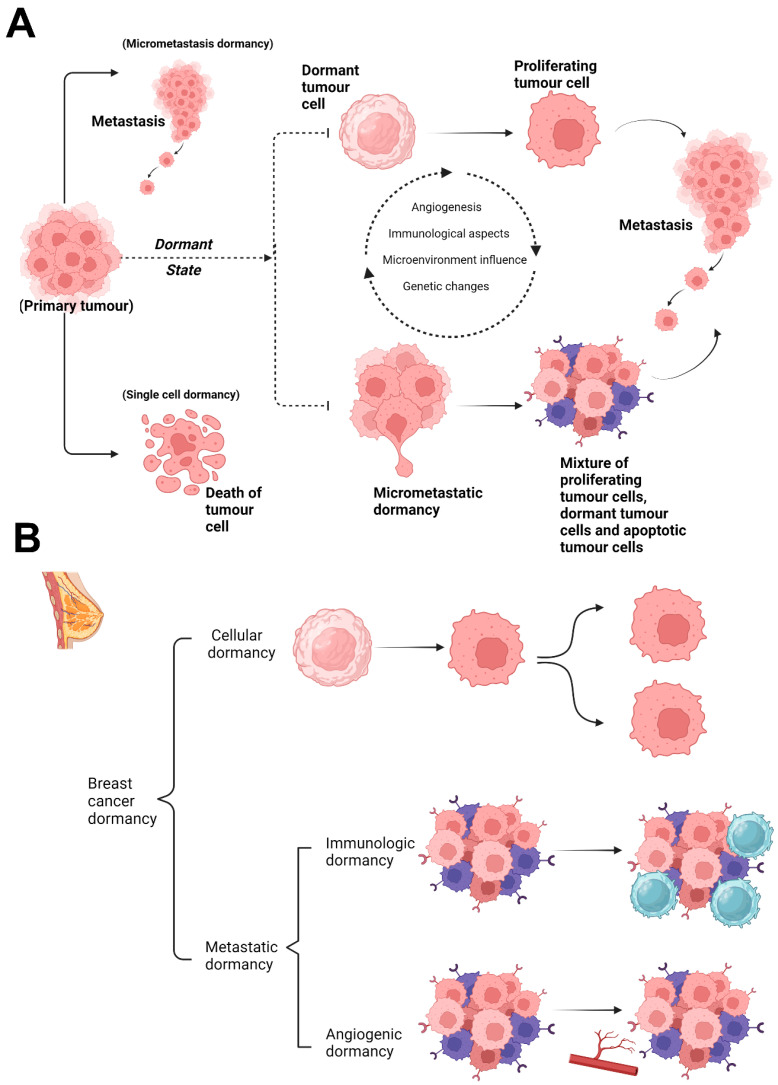
Initiating of tumour dormancy and types of dormancy in breast cancer. (**A**) The three potential pathways for breast tumour cells that break away from their primary tumour. (**B**) Biological mechanisms of hibernating breast cancer cells. Cancer dormancy can be classified physiologically into cellular dormancy and metastatic dormancy. Cancer progresses during cellular dormancy, when dormant malignant cells re-enter the cell cycle. Similarly, metastatic dormancy progresses when an angiogenic switch occurs or tumour cells avoid immune checkpoints, causing the imbalance between proliferation and death to shift towards the benefit of proliferation.

Thus, dormancy can be classified into two stages (Figure 1A) based on in vivo and in vitro models. Single dormant cells are those that have neither growth or apoptosis and are in the cell cycle arrest stage. When the microenvironment or gene expression changes, tumour cells begin to multiply [16]. The micrometastatic model describes tumour cell dormancy as a condition of balanced apoptosis and micrometastasis proliferation that does not result in a net increase in tumour mass. Growth-stimulating and inhibitory substances generated by the microenvironment, immune cells, and tumour cells themselves regulate the balance. However, the variables that influence the duration of the dormancy phase remain unknown [17].

Though they are quite different forms of dormancy, single-cell dormancy and dormant micrometastasis are both thought to be precursors of metastatic disease. These two forms of latency may coexist in the total population of dormant breast cancer in a given cancer patient; they are not mutually exclusive.

## 3. Mechanisms of Hibernating Breast Cancer Cells

As to a recent assessment, there are two broad groups into which the most current experimental models of breast cancer dormancy can be classified, each with unique growth dynamics (Figure 1B) [18]. The equilibrium between cell death and proliferation causes the first category, known as micrometastatic dormancy, to experience a halt in the overall growth of the tumour. In this group, the most common models are immunologic dormancy and angiogenic dormancy [19]. The second type, which is also known as solitary cell dormancy or single-cell dormancy, is characterised by the capacity of individual cancer cells to go into a transient cell-cycle arrest [20,21].

### 3.1. Single-Cell Dormancy

In the model of single-cell dormancy, individual tumour cells that have separated from the parent tumour reach the organ, where they will eventually metastasise and go into a protracted state of mitotic arrest. This paradigm of halted apoptosis is in contrast to the micrometastatic dormancy model, which in general describes how cell death balances proliferation in micrometastatic foci [19]. While there are a number of lines of evidence indicating the existence of dormant cancer cells, the fundamental process underpinning cellular dormancy is arguably the least understood because it is still very difficult to characterise individual cancer cells in vivo [22].

An immunohistochemistry assay (IHC), like Ki-67 or TUNEL stain, has been used as the main method to identify dormant cancer cells in vivo. However, this approach offers only a limited understanding of a dynamic process. More modern methods, like live-cell imaging, have been able to provide more details about the growth kinetics at the single-cell level in experimental models. In one study, dormant cancer cells were found to survive in mouse models of metastasis for at least 11 weeks after injection using in vivo videomicroscopy [23].

Tumour necrosis factor-related apoptosis-inducing ligand (TRAIL) receptor blockage may provide dormant tumour cells a survival advantage by causing the apoptosis to become arrested [24,25]. There are two well-documented approaches associated with TRAIL-receptor blockage that might be relevant to quiescent tumour cells. One is Osteoprotegerin, a key component of the tumour necrosis factor receptor superfamily, which has the ability to inhibit TRAIL receptors in cancer cells [26]. Remarkably, Osteoprotegerin is secreted by breast cancer patients’ bone marrow stromal cells in sufficient amounts to prevent apoptosis in vitro [27]. On the other hand, c-Src, a tyrosin-specific kinase implicated in the progression of breast cancer, has been shown to promote cancer cell survival by giving resistance to TRAIL [28,29].

### 3.2. Micrometastatic Dormancy

The hypothesis that minimal residual cancer might be classified into “active” and “dormant” groups based on favourable mutations acquired just before the emergence of a highly aggressive metastatic clone is supported by genetic data [30,31]. The dormancy of a micrometastasis appears to be brought on by a balance between cell proliferation and apoptosis as opposed to dormancy brought on by mitotic arrest, which prevents the tumour from growing larger. Proangiogenic proteins and angiogenic inhibitors generated by tumour and stromal cells, along with immunologic, hormonal, or other microenvironmental factors, control this ongoing balancing [32,33,34].

According to previous studies, more aggressive diseases are associated with the micrometastatic dormancy model, whereas indolent breast tumours may fall under the single-cell dormancy concept [35,36,37]. In fact, a set of investigations by Barkan et al. showed that, whereas oestrogen receptor (ER)-positive MCF7 remained in a state of mitotic arrest, more aggressive basal-type cell lines, like MDA-MB-231, multiplied quickly [38].

This finding may have connected the dormancy type linked to arrested growth with the less aggressive disease phenotype. However, which model more accurately reflects tumour dormancy in breast cancer is an open subject at this point in time.

#### 3.2.1. Angiogenic Dormancy

Angiogenesis is the formation of new blood vessels from existing ones, which is necessary for life and healing but also occurs in cancer and other disorders, beginning in utero and continuing into old age. John Hunter contributed the first documented scientific insights into the area of angiogenesis. His findings revealed that the relationship between vascularity and metabolic requirements exists in both states of health and illness [39,40,41,42]. However, the idea that tumours rely on the generation of new capillaries to sustain their growth was proposed in the 1970s, prompting significant interest in understanding the mechanisms beneath angiogenesis [43].

Judah Folkman proposed that the human body constantly keeps tiny tumours in control by preventing them from recruiting additional blood supplies [44]. Folkman specifically evoked angiogenic dormancy to explain the disparity between the high prevalence of undiagnosed breast tumour discovered at autopsy and the low rate at which these malignancies become clinically significant throughout a lifetime.

Among the most recent studies, Indraccolo et al. found that a short-term disruption in the tumour microenvironment, such as a transitory angiogenic burst, could be sufficient for interrupting tumour dormancy [33]. Furthermore, a set of cancer cells may remain latent in a hibernating state if the angiogenic switch is not activated, according to Naumov et al. [45]. These two studies demonstrate the key role of angiogenesis in tumour dormancy.

Therefore, a better understanding of the angiogenic process and attempts to manipulate its mechanisms during tumour dormancy could potentially help gain control over the latter.

#### 3.2.2. Immunologic Dormancy

Cancer cells confront three major hurdles during the processes of survival and proliferation. First, they must trick the immune system, which is capable of eliminating most tumours. Second, they must withstand anticancer treatments, and, third, they have to invade distant tissues and cause metastasis [46,47,48,49,50,51,52,53]. Cancer dormancy is required to address all of these difficulties. During periods of dormancy, cancer cells modify their genetic makeup and prepare for the next stage of growth. As a result, without immunological dormancy, cancer cells would be unable to survive in a new environment or develop resistance to immune system attacks.

Previous research has identified metastatic melanoma as a potential manifestation of immunological dormancy in clinical contexts. For instance, a case report was published that revealed instances where metastatic melanoma emerged 1 to 2 years following organ transplant in two recipients who received kidneys from the same donor. This donor had been treated for primary melanoma over 12 years prior to death, appearing disease-free at the time of organ donation [54].

While many observations initially suggest a link between immunological dormancy and cancer development over an extended period [55,56,57,58,59,60], it is crucial to acknowledge the inherent complexities. Recipients in such studies, like other immunosuppressed patients, exhibit heightened susceptibility to various diseases, including cancer, due to their compromised immune systems. This raises the need for further exploration and clarification.

As a result, while metastatic melanoma and its association with post-transplantation patients are not the focus of this review, the question of whether these occurrences serve as evidence of immunological dormancy and its significant role in tumour development remains open for debate. Thus, continued research efforts in this area are strongly encouraged.

## 4. Identification and Characterisation of Hibernating Breast Cancer Cells

Connecting the dots between our limited understanding of biology and the clinically observed phenomena of hibernating breast cancer cells requires functional detection, isolation, and characterisation of hibernating breast cancer cells in a physiological context, which has always been a hurdle [61,62,63,64,65]. Since dormant cells, in general, lack a known specific marker, existing isolation techniques that rely on enriching tumour cells using epithelial surface proteins may miss the target population, particularly if dormant cells have the potential to become stem cells [66].

Research on this process in tissue culture is obviously limited, but there have been advancements made to increase the applicability of in vitro studies. For instance, three-dimensional culture systems made of basement membrane components more closely resemble in vivo extracellular matrix components, facilitating more accurate studies of cellular behaviour in conditions that mimic the physiological environment [67,68]. Additionally, directly engineering microenvironmental niches has shown promise in replicating the complex interactions that occur within the human body [38,69,70], further enhancing the relevance of in vitro models [67,68].

Recent advancements in single-cell analysis technologies are crucial for making tangible progress in comprehending hibernating breast cancer cells. Techniques such as single-cell RNA sequencing (scRNA-seq) and single-cell DNA sequencing (scDNA-seq) have been pivotal in uncovering the heterogeneity of cancer cell populations and identifying rare dormant cells [71,72,73,74]. Utilising micromanipulation, combined with transcriptome or whole-genome amplification phases and miniaturised PCR methodologies, has enabled the detailed analysis of individual cells, providing insights into the genetic and epigenetic landscapes of dormant cancer cells [71,72,73,74].

Furthermore, the development of advanced imaging techniques, such as intravital microscopy, has allowed researchers to observe dormant cells in real-time within living organisms, providing invaluable data on the behaviour and characteristics of these cells in their native environment [75,76]. This approach, combined with sophisticated bioinformatics tools, has significantly enhanced our ability to detect, isolate, and characterise hibernating breast cancer cells.

Bridging the gap between biological understanding and clinical observations of hibernating breast cancer cells involves integrating advanced detection and analysis technologies with innovative culture systems. Continued advancements in these areas are essential for unravelling the complexities of cancer cell dormancy and developing effective therapeutic strategies.

## 5. Potential Therapeutic Strategies Targeting Hibernating Breast Cancer Cells

Our knowledge of hibernating breast cancer cells must change from being solely based on clinical observations to include a thorough understanding of the underlying biological mechanisms [77,78,79,80,81,82,83]. This is because the insights gained will have significant consequences in regard to managing the disease for a number of reasons.

Improvements in overall and relapse-free survival have been achieved with recent developments in the field of systemic chemotherapy. Chemotherapy works best against rapidly proliferative cells. However, hibernating breast cancer cells are primarily either slowly proliferating or in a condition of halted growth, which explains why certain breast cancer patients do not respond well to typical cytotoxic regimens and why alternative treatment approaches are required. Furthermore, the decisive ability of a substantial number of hibernating breast cancer cells to display characteristics that are stem cell-like, including immunophenotype, low proliferation rate, and growth characteristics, may pose a significant challenge to cytostatic therapy [84,85].

**Figure 2:**
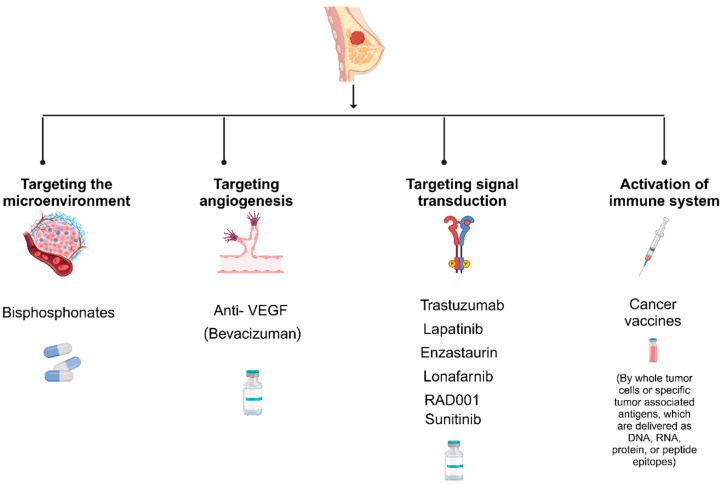
Potential therapeutic strategies targeting hibernating breast cancer cells. (1) Targeting the microenvironment. (2) Targeting angiogenesis. (3) Targeting signal transduction. (4) Activation of the immune system. Hormone therapy primarily targets actively proliferating cancer cells by blocking hormone receptors and slowing the growth of hormone-sensitive tumours. However, hibernating breast cancer cells are in a quiescent state, rendering them less responsive to hormone therapy, which relies on active-cell division to be effective.

Therefore, research and studies on tumour dormancy in general, and hibernating breast cancer cells in particular, might contribute positively towards determining whether the disease in that particular group of breast cancer patients is dormant and what type of dormancy mechanism is active, be it cellular or metastatic. Based on recent studies focusing on tumour dormancy [86,87,88,89,90,91,92], potential therapeutic strategies include (1) targeting the microenvironment, (2) targeting angiogenesis, (3) targeting signal transduction, and (4) activating the immune system (Figure 2). Current evidence suggests that hormone therapy primarily targets actively proliferating cancer cells by blocking hormone receptors and slowing the growth of hormone-sensitive tumours. However, hibernating breast cancer cells are in a quiescent state, rendering them less responsive to hormone therapy, which relies on active-cell division to be effective. Moreover, the unique biology of hibernating cells, which often evade conventional therapies, necessitates the exploration of al-ternative strategies that directly target their dormant nature. Therefore, the figure emphasizes therapies specifically designed to awaken or eliminate these dormant cells rather than hormone therapies that are not directly effective in this context. This approach is supported by recent studies which indicate the need for targeted treatments that address the distinct characteristics of hibernating cancer cells [93,94,95,96].

### 5.1. Targeting the Microenvironment

Bisphosphonates [97,98,99,100], which are strong inhibitors of osteoclast-mediated bone resorption, are one option for treatment [101,102,103]. There is growing evidence of their powerful anticancer action both in vivo and in vitro, implying that these medicines have a role beyond their traditional application in the treatment of bone metastases consequent to breast cancer [104,105,106]. Several studies have demonstrated their efficacy in the prevention of bone metastases as well as their positive effects on survival in specific patient subgroups [107,108,109,110,111,112]. These elements are achieved mostly through inhibiting tumour cell adhesion, invasion, and proliferation; inducing apoptosis; and affecting the microenvironment through modified growth factor and cytokine release.

Cancer research has recently placed more emphasis on the intricate relationships that exist between cancerous cells and their surroundings, as well as the cancer cell itself. The degree of angiogenesis, invasion, and survival of cells is determined by the particular microenvironment. As such, the surrounding microenvironment should be targeted in addition to the tumour cell during systemic treatment.

### 5.2. Targeting Angiogenesis

Patients with metastatic breast cancer have demonstrated encouraging outcomes using bevacizumab, a recombinant humanised monoclonal antibody against vascular endothelial growth factor (VEGF), when administered with chemotherapy [113,114,115,116,117,118]. Although antiangiogenic drugs like bevacizumab have shown promise in clinical trials, it is important to note that they have not received approval specifically for the treatment of breast cancer.

Blocking the “angiogenic switch”, which is the angiogenic activation of growth progression, antiangiogenic medicines may potentially help in managing dormant tumour cells [119]. The growth and survival of dormant tumour cells rely on the formation of a vascular bed and the recruitment of blood vessels once they emerge from their dormant state and reach a particular tumour mass. Therefore, the suppression of angiogenesis remains a possible therapeutic approach and a crucial aspect of tumour progression research.

Despite the lack of specific approval for breast cancer, ongoing research and clinical trials continue to explore the potential of antiangiogenic therapies in various cancer types. Understanding and targeting the mechanisms of angiogenesis could lead to significant advancements in the treatment of breast cancer in the future.

### 5.3. Targeting Signal Transduction

Trastuzumab is a monoclonal antibody that targets the HER2 receptor’s extracellular domain. Adjuvant trials with Trastuzumab in breast cancer patients demonstrated remarkable outcomes that featured much lower recurrence rates [120,121,122,123]. The most recent studies [124,125,126,127] have revealed that the combination of neoadjuvant pertuzumab and trastuzumab alongside chemotherapy leads to elevated rates of pathological complete response (pCR) in real-world patients with HER2-positive early breast cancer, demonstrating a satisfactory safety record.

Cell-modulating medications, such as antibodies or small compounds, are increasingly targeting specific cell cycle and cancer targets. The HER2 receptor is likely the primary target in such approaches [128]. The growth factor receptor plays a crucial role in cell growth, differentiation, and survival. Regardless of the initial HER2-negative status of the primary tumour, HER2 gene amplification can occur during disease progression [129,130,131]. Thus, patients who are not eligible for HER2-based treatment because their tumours are HER2-negative but who have HER2-positive minimal residual disease (MRD) may benefit from this therapeutic approach.

Therefore, a personalised targeted treatment may be possible if it becomes feasible to identify the specific nature of MRD cells and track changes in their immunophenotype and genotype as the disease progresses. However, it is important to remember that eliminating of dormant tumour cells might not directly affect the prognosis for survival. Prospective randomised trials are needed to determine whether these medicines are beneficial for patients with chronic MRD.

### 5.4. Activating the Immune System

Cancer vaccines are therapeutic approaches intended to activate the patient’s immune system, prompting it to identify and combat cancer cells [132,133,134]. In contrast to traditional vaccines that prevent infectious diseases, cancer vaccines are developed to address existing cancers by leveraging the body’s immune response to target and eliminate cancer cells or impede their proliferation [135,136]. These vaccines may comprise tumour-associated antigens, peptides, proteins, or even intact whole tumour cells, as seen in breast cancer, combined with adjuvants to bolster immune activity [137,138,139]. The objective is to educate the immune system to perceive cancer cells as being foreign or aberrant, empowering it to launch a precise and potent attack against the tumour.

**Figure 3:**
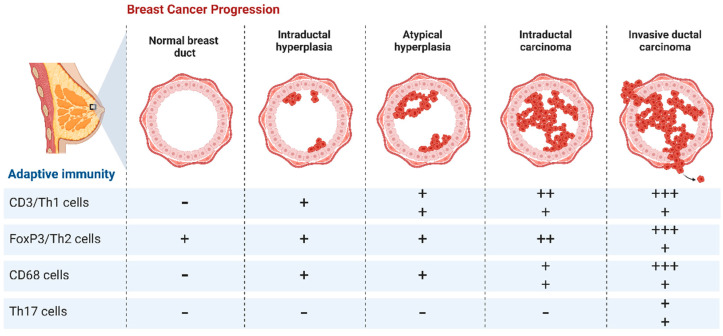
Adaptive immunity during breast cancer progression.

As interesting as this therapeutic strategy sounds, i.e., using therapeutic cancer vaccines to stimulate an adaptive immune response against tumours (Figure 3), its clinical activity remains restricted and discouraged [140,141]. It is worth mentioning that the vaccinations are evaluated on patients with advanced metastatic disease, which is typically resistant to other conventional treatments, which may account for these unfavourable results. Patients with dormant MRD may benefit more from therapeutic vaccinations because the target-to-effector ratio is more favourable in these patients. There are not many adjuvant trials underway at this moment.

## 6. Evolution and Cutting-Edge Developments in Hormone-Targeted Therapy and Immunotherapeutics for Breast Cancer

### 6.1. Historical Perspective and Breakthroughs in Hormone-Targeted Therapy

The treatment of breast cancer has evolved significantly over the decades, particularly with the advent of hormone-targeted therapies. Historically, the understanding that estrogen plays a crucial role in the proliferation of breast cancer cells has laid the foundation for developing therapies targeting the estrogen receptor (ER). Tamoxifen, introduced in the 1970s, was the first selective estrogen receptor modulator (SERM) and marked a significant breakthrough, reducing recurrence rates in ER-positive breast cancer patients by approximately 40–50% [142,143].

In the 1990s, aromatase inhibitors (AIs) such as anastrozole, letrozole, and exemestane emerged, offering an alternative for postmenopausal women by inhibiting the enzyme aromatase, responsible for estrogen synthesis. These inhibitors showed superior efficacy in reducing cancer recurrence compared to tamoxifen in certain patient groups [144,145]. More recently, selective estrogen receptor degraders (SERDs) like fulvestrant have been developed. Fulvestrant not only blocks the estrogen receptor but also promotes its degradation, offering another line of defence against hormone-receptor-positive breast cancer. Clinical trials have demonstrated its effectiveness, especially in patients who have progressed onto other hormonal therapies [146,147].

### 6.2. Recent Advances in Immunotherapeutics

Immunotherapy has revolutionised the treatment landscape for many cancers, including breast cancer. The most significant progress has been in the treatment of triple negative breast cancer (TNBC), a subtype that lacks ER, PR, and HER2 receptors, making it unresponsive to hormonal therapies and HER2-targeted treatments. Checkpoint inhibitors like pembrolizumab (Keytruda) represent a significant advancement. Pembrolizumab targets the PD-1/PD-L1 pathway, effectively enhancing the immune system’s ability to recognise and destroy cancer cells. Studies have shown that, when used in combination with chemotherapy, pembrolizumab significantly improves the overall survival rates in TNBC patients [146,148].

Moreover, PARP inhibitors such as olaparib and talazoparib have shown promise, particularly in patients with BRCA1/2 mutations. These drugs exploit the DNA repair weaknesses in cancer cells, leading to cell death. Clinical trials have demonstrated the efficacy of olaparib in prolonging progression-free survival in HER2-negative metastatic breast cancer patients with BRCA mutations [149].

### 6.3. Most Recent Innovations

Innovations in targeted therapy continue to evolve. The development of CDK4/6 inhibitors, like palbociclib, ribociclib, and abemaciclib, represents a leap forward in treating hormone-receptor-positive breast cancer. These drugs work by inhibiting cyclin-dependent kinases 4 and 6, which are crucial for cell cycle progression. When combined with hormonal therapies, CDK4/6 inhibitors have been shown to significantly improve progression-free survival [150,151]. Additionally, new combinations of therapies are being explored. For instance, the combination of the FGFR inhibitor futibatinib with fulvestrant has shown promising antitumor activity in patients with advanced HR-positive breast cancer harbouring FGFR1 amplifications [152].

On the immunotherapy front, combination strategies involving checkpoint inhibitors and other agents are under investigation. These include combinations with PARP inhibitors and other novel agents aiming to overcome resistance mechanisms and improve therapeutic outcomes.

## 7. Epigenetic Regulation of Tumour Dormancy

Epigenetic modifications, particularly alterations in chromatin structure, have emerged as pivotal regulators of tumour dormancy. Understanding the role of chromatin modifications in this process is essential for developing targeted therapeutic strategies.

### 7.1. DNA Methylation

**Figure 4:**
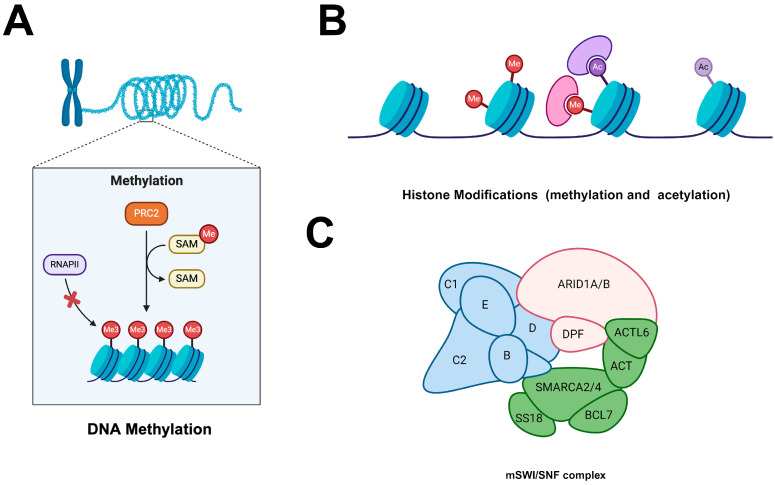
The potential epigenomic role in breast cancer dormancy (**A**) DNA methylation; (**B**) Histone modifications (methylation and acetylation); (**C**) chromatin remodelling complexes.

DNA methylation, as illustrated in (Figure 4), involves the addition of methyl groups to cytosine residues within CpG dinucleotides, a process catalysed by DNA methyltransferases (DNMTs). This epigenetic modification has profound effects on the transcriptional status of genes, playing a critical role in the regulation of tumour dormancy [153].

In the context of gene promoters, hypermethylation can lead to the silencing of tumour suppressor genes. Tumour suppressor genes are essential for controlling cell growth, promoting DNA repair, and inducing apoptosis in damaged cells. When these genes are silenced through promoter hypermethylation, cells can escape growth inhibition and evade apoptosis, contributing to the establishment of a dormant state. This silencing ensures that the cells do not proliferate under unfavourable conditions, thereby aiding in their survival until conditions become conducive for growth [154].

Conversely, hypomethylation of specific genomic regions, including gene promoters and repetitive elements, can result in the activation of oncogenes. Oncogenes are genes that, when overexpressed or mutated, drive the proliferation and survival of cancer cells. Hypomethylation can reactivate these oncogenes, leading to the re-entry of dormant tumour cells into the cell cycle and promoting tumour progression and metastasis [155]. Moreover, global DNA hypomethylation can contribute to genomic instability, further facilitating cancer cell evolution and adaptation.

DNA methylation patterns are not static and can be dynamically regulated in response to various signals, including environmental stress, therapeutic interventions, and cellular signalling pathways. The interplay between hypermethylation and hypomethylation at different genomic loci determines the balance between dormancy and the reactivation of tumour cells. For instance, in a dormant state, tumour cells may exhibit a methylation pattern that represses genes involved in cell proliferation while maintaining the expression of genes necessary for cell survival and stress resistance. Upon receiving appropriate signals, such as changes in the tumour microenvironment or exposure to specific growth factors, the methylation landscape can shift, leading to the reactivation of proliferative genes and the exit from dormancy [156].

The regulatory influence of DNA methylation on gene expression is mediated through several mechanisms. Methylation of CpG islands in gene promoters can directly prevent the binding of transcription factors, thereby blocking gene transcription. Additionally, methylated DNA can recruit methyl-CpG-binding domain (MBD) proteins, which in turn attract histone deacetylases (HDACs) and other repressive chromatin modifiers. This recruitment results in a more compact and inaccessible chromatin structure, further silencing gene expression.

Understanding the intricate details of DNA methylation and its impact on gene expression is crucial for developing therapeutic strategies aimed at targeting tumour dormancy and preventing cancer recurrence. Drugs that inhibit DNMTs, such as azacytidine and decitabine, are already in use for treating certain haematological malignancies and have the potential to reactivate silenced tumour suppressor genes. Additionally, combining DNA methylation inhibitors with other epigenetic therapies, such as histone deacetylase inhibitors or chromatin remodelling agents, may provide a synergistic approach to disrupting the epigenetic mechanisms maintaining tumour dormancy and reduce the risk of tumour reactivation and metastasis.

### 7.2. Histone Modifications

Histone modifications, depicted in (Figure 4A,B), encompass a variety of chemical changes to histone proteins, including methylation, acetylation, phosphorylation, and ubiquitination. These modifications play a crucial role in dynamically regulating chromatin structure and gene expression, making them fundamental to numerous cellular processes [157,158].

Methylation involves the addition of methyl groups to histone proteins, with trimethylation of histone H3 lysine 4 (*H3K4me3*) being a prominent mark associated with the activation of transcription. This specific modification is often found at the promoters of actively transcribed genes, where it facilitates the recruitment of transcriptional machinery and enhances gene expression. Conversely, trimethylation of histone H3 lysine 27 (*H3K27me3*) serves as a repressive mark that is commonly located at gene promoters and enhancers associated with transcriptional repression. This modification is crucial for maintaining gene silencing during development and differentiation [159].

Acetylation, another significant histone modification, typically occurs at lysine residues and is catalysed by histone acetyltransferases (HATs). Acetylation neutralises the positive charge of histones, leading to a more relaxed chromatin structure that permits transcriptional activation. Histone deacetylases (HDACs) reverse this process, removing acetyl groups and promoting chromatin compaction, thereby repressing gene expression.

Phosphorylation involves the addition of phosphate groups to serine, threonine, or tyrosine residues of histones. This modification is often associated with chromatin remodelling and can influence both transcriptional activation and repression, depending on the specific context and residues involved. Phosphorylation is also pivotal during cell cycle progression and DNA damage response, highlighting its dynamic role in chromatin regulation [157]. Ubiquitination, the addition of ubiquitin molecules to histones, can signal for histone degradation or modulate chromatin structure and function. This modification is involved in various DNA-related processes, including transcription, repair, and replication.

Alterations in histone modification patterns significantly contribute to the establishment and maintenance of tumour dormancy. Tumour cells can modulate histone marks to induce a state of cell cycle arrest, allowing them to survive in a quiescent state under unfavourable conditions. For instance, the enrichment of repressive marks such as *H3K27me3* at proliferation-associated genes can maintain these genes in an inactive state, preventing cell division. Conversely, activating marks like *H3K4me3* at genes involved in cell survival and stress responses can promote the expression of factors that are necessary for the dormant cells to withstand adverse environments [160]. Thus, the precise orchestration of histone modifications is crucial for the regulation of gene expression patterns that underpin tumour dormancy and persistence.

### 7.3. Chromatin Remodelling

Chromatin remodelling complexes, illustrated in Figure 4B, include key players such as the SWI/SNF and ISWI complexes. These complexes are essential in regulating access to DNA by altering the positioning and composition of nucleosomes, which are the fundamental units of chromatin structure consisting of DNA wrapped around histone proteins [161,162]. By remodelling nucleosomes at gene regulatory regions, these chromatin remodelers facilitate or hinder the accessibility of transcription factors and RNA polymerase, directly influencing gene expression. This regulation is crucial for maintaining the dormant state of tumour cells, as it ensures the appropriate genes are either activated or repressed to sustain dormancy [163].

The SWI/SNF complex, for example, utilises the energy derived from ATP hydrolysis to reposition or evict nucleosomes, thus exposing or occluding specific DNA sequences. This action can lead to the activation of tumour suppressor genes or the repression of oncogenes, contributing to the maintenance of cellular quiescence and preventing unchecked cell proliferation. Similarly, the ISWI complex fine-tunes nucleosome spacing, which is vital for the orderly organisation of chromatin and the precise regulation of gene expression.

The genesis and persistence of cancer latency are largely determined by a complex interplay of epigenetic regulations involving DNA methylation, histone modifications, and chromatin remodelling. DNA methylation, typically occurring at cytosine residues in CpG islands, is a well-known mechanism for gene silencing. Aberrant methylation patterns can lead to the repression of tumour suppressor genes, aiding in the establishment of a dormant cancer state. Conversely, demethylation of certain genomic regions can reactivate genes necessary for cell survival under stress, contributing to the persistence of dormant tumour cells [162].

Histone modifications, such as methylation, acetylation, phosphorylation, and ubiquitination, add another layer of regulation. These modifications can alter chromatin structure and recruit specific effector proteins that either activate or repress transcription. For instance, *H3K4me3* is associated with gene activation while *H3K27me3* is linked to gene repression. These histone marks, by influencing chromatin dynamics, play pivotal roles in regulating gene expression programs that govern cell cycle arrest and survival during dormancy [161]. Chromatin remodelling complexes integrate these epigenetic signals by repositioning nucleosomes in response to histone modifications and DNA methylation patterns. This dynamic restructuring of chromatin facilitates the appropriate response to environmental cues, ensuring that tumour cells can remain in a dormant state or reactivate when conditions become favourable.

Understanding how these epigenetic mechanisms work together to influence gene expression programs is essential for designing therapeutic strategies aimed at blocking tumour reactivation and metastasis. By targeting specific components of DNA methylation pathways, histone modification enzymes, or chromatin remodelling complexes, it may be possible to disrupt the epigenetic landscape that supports tumour dormancy. Such interventions could prevent dormant cancer cells from re-entering the cell cycle and forming metastatic lesions, thereby improving long-term cancer outcomes.

## 8. The Epigenetic Influence of G9a Enzyme in Breast Cancer Relapse and Most Recent Therapeutic Insights

Cancer has long been recognised as a genetic illness; somatic mutations in DNA contribute to cell immortalisation and loss of intercellular adhesion, which result in the invasion and metastasis elements that are the histological hallmarks of cancer [164]. However, cancer is a complex disease caused by epigenetic changes in normal cells. Breast cancer development is highly linked to aberrant epigenetic manipulation [165]. As a result of their reversibility, contemporary therapeutic techniques focus on epigenetic alterations rather than genetic mutations.

The rapidly growing field of epigenetics investigates the molecules and biological mechanisms that, within the confines of the same DNA sequence, can sustain distinct gene activity states [166]. Epigenetic mechanisms play vital roles in controlling gene transcription; maintaining genomic stability; and ensuring normal cell growth, development, and differentiation. Unlike genetic alterations, epigenetic changes do not modify the underlying genetic material or reprogram genomic information [167]. Three primary mechanisms responsible for these alterations include DNA methylation, histone modification, and non-coding RNAs (ncRNAs) [168]. These mechanisms serve as essential regulators of cellular immunity by modulating gene expression and transcription within distinct cells and tissues.

Since recurrence of breast cancer remains a pressing concern in clinical oncology, requiring a deeper understanding of the molecular pathways driving disease progression post-treatment. Rosano et al. recently highlighted the role of epigenetic modifications, particularly alterations in histone methylation, has been recently highlighted in regard to contributing to cancer recurrence [169]. Among these epigenetic regulators, G9a histone methyltransferase has garnered attention for its involvement in breast cancer recurrence.

G9a is known to methylate histone H3 at lysine 9 (*H3K9me*), resulting in chromatin compaction and transcriptional repression of tumour suppressor genes. This epigenetic modification promotes cancer cell proliferation, invasion, and metastasis, thereby facilitating the recurrence of breast cancer following initial treatment [170]. Additionally, G9a is implicated in maintaining the stem-like properties of cancer cells, which are associated with tumour initiation, progression, and therapy resistance. By regulating genes associated with stemness, G9a sustains the survival and aggressiveness of breast cancer cells, contributing to disease relapse post-therapy [171,172]. Furthermore, G9a-mediated epigenetic alterations have been linked to the induction of epithelial-to-mesenchymal transitioning (EMT), a process that is critical for cancer cell dissemination and metastasis [173]. Through the modulation of gene expression involved in EMT, G9a enhances the invasive and migratory capabilities of breast cancer cells, promoting the establishment of recurrent lesions at distant sites.

Targeting G9a therapeutically presents a promising approach to mitigate breast cancer recurrence and improve patient outcomes. Several small-molecule inhibitors of G9a have demonstrated efficacy in preclinical models, underscoring the potential for pharmacological intervention to disrupt G9a-mediated oncogenic pathways. However, challenges such as non-specific effects and the emergence of resistance necessitate the further refinement of G9a-targeted therapies for clinical use.

Researchers at The Institute of Cancer Research in London have recently employed hormone therapy in their most recent trials to suppress the G9a enzyme, which catalyses *H3K9me2*, thereby preventing cancer cells from going into dormancy as a result of epigenetic modifications [169]. Reduced expression of the G9a enzyme was also identified in a cohort investigation, which found that it can greatly reduce the possibility of a recurrence of breast cancer. The importance of regulating epigenetic modifications in complicated behaviour in cancer has likely been confirmed by this study.

This early stage study revealed that breast cancer cells adopt an inactive state to evade therapy, contrasting the common tendency for cancer to adapt and evolve to resist treatment. Despite the valuable contribution this study makes to understanding epigenetic modifications and their potential as targeted therapy, it is crucial not to overlook the complex nature of epigenetic changes. They are transient by nature and more susceptible to rapid alteration than the fixed DNA code; thus, there is still much more to learn.

## 9. Emerging Biomarkers in Breast Cancer

**Figure 5:**
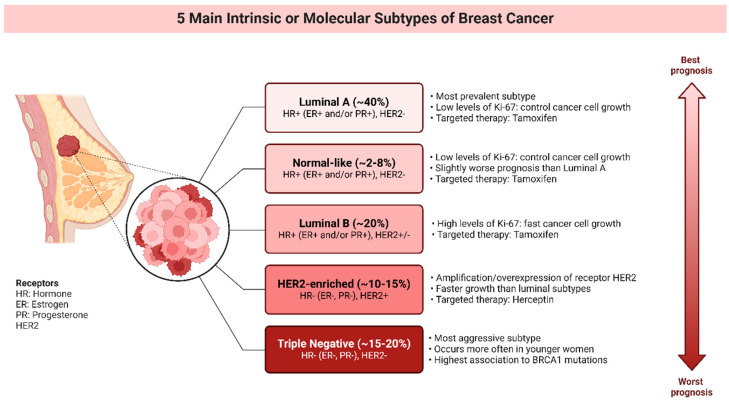
Molecular subtypes of breast cancer.

Although traditional biomarkers like estrogen receptor (ER), progesterone receptor (PR), and human epidermal growth factor receptor 2 (HER2) have guided clinical decisions (Figure 5), recent advancements in molecular profiling have brought to light promising novel biomarkers for breast cancer diagnoses, prognoses, and treatment response predictions.

### 9.1. Circulating Tumour Cells (CTCs)

Circulating tumour cells (CTCs), released from the primary tumour into the bloodstream, hold significant promise as biomarkers for the early detection of cancer, monitoring disease progression, and predicting treatment responses [174]. These cells, which break away from the primary tumour mass and enter the circulatory system, can provide a wealth of information about the molecular characteristics of the tumour and its potential to metastasise.

The detection and characterisation of CTCs have been greatly advanced by various sophisticated technologies. Microfluidic-based approaches use specially designed devices with microscopic channels to separate CTCs from other blood components based on their unique physical and biochemical properties. These microfluidic devices can capture CTCs with high efficiency and purity, enabling detailed analysis of their genetic and phenotypic characteristics.

Immunomagnetic separation is another widely used technique for isolating CTCs. This method involves the use of magnetic nanoparticles coated with antibodies that specifically bind to antigens expressed on the surface of CTCs. Once bound, a magnetic field is applied to separate the CTCs from the rest of the blood cells. This technique is highly specific and allows for the enrichment of CTCs, making it easier to study their properties and behaviour [175,176].

The analysis of CTCs can provide critical insights into the metastatic potential of cancer. By examining the genetic mutations, gene expression profiles, and protein markers of CTCs, researchers can identify key characteristics that enable these cells to survive in the bloodstream, evade the immune system, and establish secondary tumours in distant organs. For instance, CTCs that express epithelial–mesenchymal transition (EMT) markers may be more capable of invading new tissues and forming metastases. This can be used further to assess the aggressiveness of the cancer and tailor treatment strategies accordingly [177].

Moreover, CTCs offer a non-invasive means of monitoring disease progression and therapeutic resistance. Traditional biopsy methods involve the surgical removal of tumour tissue, which can be invasive, painful, and risky for the patient. In contrast, a “liquid biopsy” involving the analysis of CTCs from a blood sample is much less invasive and can be performed repeatedly over time. This allows clinicians to track changes in the tumour’s molecular profile, monitor how the cancer responds to treatment, and detect the emergence of resistance mechanisms [176].

For example, if CTC analysis reveals the presence of specific genetic mutations associated with resistance to a particular therapy, clinicians can adjust the treatment plan to include alternative drugs that are more likely to be effective. Additionally, monitoring the number of CTCs in the bloodstream can provide an indication of how well the treatment is working. A decrease in CTC count may suggest that the therapy is effectively targeting the tumour, while an increase could indicate disease progression or recurrence [177].

### 9.2. Liquid Biopsies

Liquid biopsies, a non-invasive approach, detect tumour-derived components such as circulating tumour DNA (ctDNA), circulating microRNAs, and exosomes in bodily fluids [178,179]. These biomarkers provide real-time insights into tumour heterogeneity, clonal evolution, and treatment response dynamics, which are crucial for personalised disease management [180].

ctDNA, fragments of DNA released by tumour cells into the bloodstream, reflect the genetic landscape of the tumour, allowing for the detection of mutations, copy number variations, and epigenetic alterations. This information can reveal tumour burden, track the emergence of resistance mutations, and monitor minimal residual disease. Circulating microRNAs, small non-coding RNA molecules, regulate gene expression and are often dysregulated in cancer. Their presence in blood can serve as a diagnostic and prognostic tool, offering insights into the tumour’s molecular environment and potential therapeutic targets [181].

Exosomes, small vesicles secreted by tumour cells, contain proteins, lipids, and nucleic acids that mirror the tumour’s molecular profile. Analysing exosomes can uncover the mechanisms of tumour progression, immune evasion, and metastasis, aiding in the identification of novel biomarkers and therapeutic strategies [180].

By integrating these biomarkers in liquid biopsies, it may enable a comprehensive view of the tumour’s molecular dynamics and improve patient outcomes. This approach supports the shift towards precision medicine, where treatments are tailored based on the individual molecular characteristics of each cancer patient.

### 9.3. Gene Expression Signatures

Gene expression profiling, a cutting-edge technique in oncology, plays a pivotal role in the molecular sub-classification of early stage breast cancer, enabling personalised treatment strategies [182]. This sophisticated approach involves the analysis of the expression levels of numerous genes simultaneously to derive a comprehensive molecular signature of the tumour. Notable assays in this domain include Oncotype DX, MammaPrint, and Prosigna, each providing unique insights that guide clinical decision making for early stage disease [183].

Oncotype DX, for instance, evaluates the expression of 21 genes to produce a Recurrence Score. This score helps determine the likelihood of breast cancer recurrence and the probable benefit of chemotherapy in patients with early stage, hormone-receptor-positive, HER2-negative breast cancer. Integrating data on genes associated with proliferation, hormone receptor status, and immune response enables oncologists to tailor any adjuvant therapy more precisely [184].

MammaPrint, another prominent assay, examines the activity of 70 genes to classify breast cancer into low-risk or high-risk categories for metastasis. This assay is particularly valuable for patients with early stage breast cancer regardless of hormone receptor status. By identifying patients who can safely avoid chemotherapy, MammaPrint aids in minimising overtreatment and associated toxicities, thus improving the quality of life for many patients [183].

Prosigna (PAM50), yet another important panel utilising gene expression tests, assesses the expression of 50 genes to classify breast cancer into one of four intrinsic subtypes: Luminal A, Luminal B, HER2-enriched, and Basal-like (Figure 5). This classification not only provides prognostic information but also predicts the benefit of specific therapies, enhancing personalised treatment approaches. Prosigna also generates a Risk of Recurrence (ROR) score, which further aids in determining the necessity and intensity of adjuvant treatment for early stage disease [183,185].

These gene expression signatures are instrumental in advancing breast cancer treatment by enabling more accurate risk stratification and personalised therapeutic decisions in regard to early stage disease. They assess the biological pathways, offering a view of the tumour’s behaviour. This molecular insight is crucial for identifying which patients are likely to benefit from chemotherapy, hormonal therapies, or targeted treatments, thereby optimising outcomes and minimising unnecessary interventions [183].

However, it is important to note that, while these gene expression platforms are valuable for predicting early recurrence and guiding treatment in early stage breast cancer, they have not been shown to be as effective in predicting late relapse. Ongoing research is needed to develop and validate biomarkers that can better predict long-term outcomes and late relapse [186].

Utilisation of gene expression profiling through assays like Oncotype DX, MammaPrint, and Prosigna represents a significant leap forward in the precision medicine landscape of breast cancer. These tools harness the power of genomics to provide actionable insights that are reshaping the management of early stage breast cancer, making treatment more effective and personalised [182,183,184,185].

### 9.4. Tumour Microenvironment

The tumour microenvironment, a complex and dynamic entity, plays a pivotal role in breast cancer progression and response to therapy [186]. This microenvironment is composed of a variety of cells, including immune cells, fibroblasts, endothelial cells, and extracellular matrix components, all interacting with tumour cells to influence cancer behaviour and treatment outcomes. Among the critical elements within this milieu are immune checkpoint markers such as programmed death-ligand 1 (PD-L1) and tumour-infiltrating lymphocytes (TILs) [187].

PD-L1 expression on tumour cells and immune cells can inhibit the anti-tumour immune response by binding to the PD-1 receptor on T cells, leading to immune evasion. This mechanism is exploited by cancer cells to avoid destruction by the immune system. The presence of PD-L1, therefore, serves as both a prognostic indicator, often associated with poorer outcomes, and a predictive biomarker for response to immunotherapy, particularly immune checkpoint inhibitors such as pembrolizumab and atezolizumab [188]. These therapies work by blocking the interaction between PD-1 and PD-L1, thereby reactivating the immune system to target and destroy cancer cells.

TILs are another crucial component of the tumour microenvironment. TILs, which include T cells, B cells, and natural killer (NK) cells, reflect the host immune response to the tumour. A high density of TILs has been correlated with better prognosis in breast cancer, particularly in triple negative and HER2-positive subtypes. Moreover, the presence of TILs can also predict response to immunotherapies and traditional treatments such as chemotherapy [187,188].

The integration of these novel biomarkers into clinical practice is transforming breast cancer management. By combining traditional prognostic factors, such as tumour size, grade, and hormone receptor status, with molecular and genomic insights, clinicians can develop personalised treatment strategies that are tailored to the unique biological characteristics of each patient’s cancer. This approach not only enhances the precision of risk stratification but also optimises therapeutic decision-making, thereby improving patient outcomes [188,189].

For instance, in patients with high PD-L1 expression, the addition of immune checkpoint inhibitors to the treatment regimen can significantly improve response rates and survival outcomes. Similarly, assessing TILs can help in identifying patients who are likely to benefit from more aggressive or targeted therapies. This holistic approach ensures that patients receive the most effective treatments while minimising unnecessary interventions and their associated side effects [186].

Continued research in this field is essential to identify additional biomarkers that can further refine risk stratification and therapeutic decision making. The inclusion of immune checkpoint markers like PD-L1 and TILs as prognostic and predictive biomarkers represents a significant advancement in understanding tumour cell dormancy and associated factors [187,188,189].

## 10. Innovative Bispecific Antibodies: A Promising Approach in Breast Cancer Therapy

Bispecific antibodies (BsAbs) are a cutting-edge class of therapeutic agents designed to target two distinct antigens or epitopes simultaneously, thereby enhancing the precision and effectiveness of cancer treatments. This innovative approach is particularly promising for breast cancer. BsAbs can specifically target tumour-associated antigens and critical molecular pathways, improving therapeutic outcomes by engaging the immune system and directing it to attack cancer cells more effectively.

**Figure 6:**
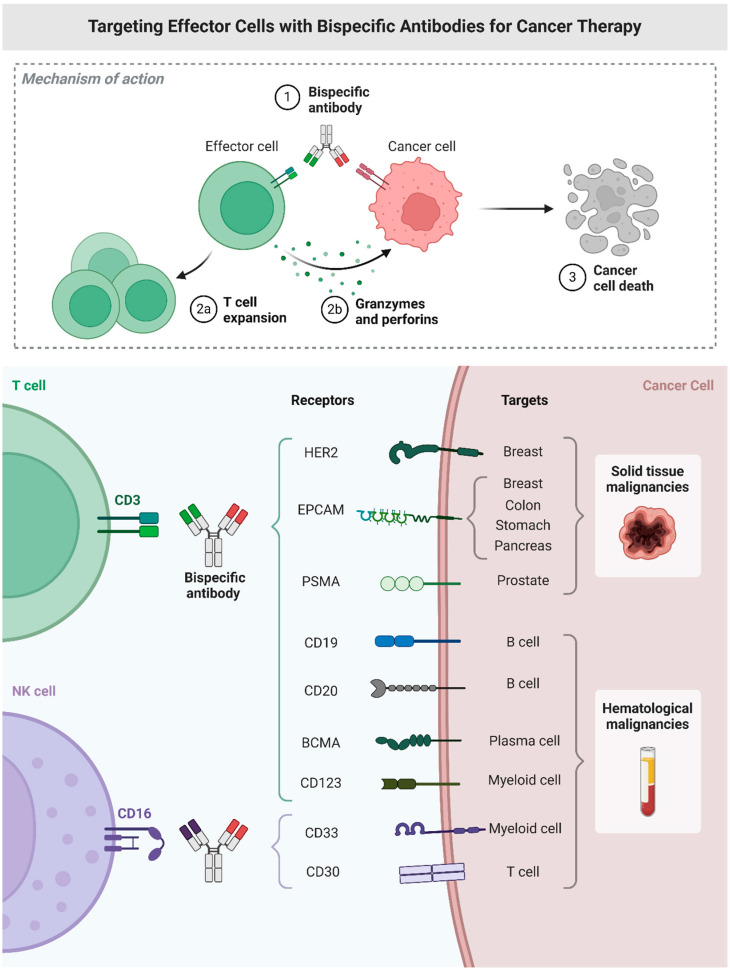
Targeting effector cells with bispecific antibodies for cancer therapy.

BsAbs function by linking two distinct antigens (Figure 6), typically a tumour-associated antigen (TAA) and an immune effector cell, thereby facilitating a targeted immune response against cancer cells. One arm of the BsAb binds to a TAA expressed in breast cancer cells, such as HER2, while the other arm binds to a receptor in immune cells, such as CD3 in T cells. This dual engagement leads to the activation of immune cells directly at the tumour site, enhancing cytotoxic activity and reducing off-target effects [190,191].

**Figure 7:**
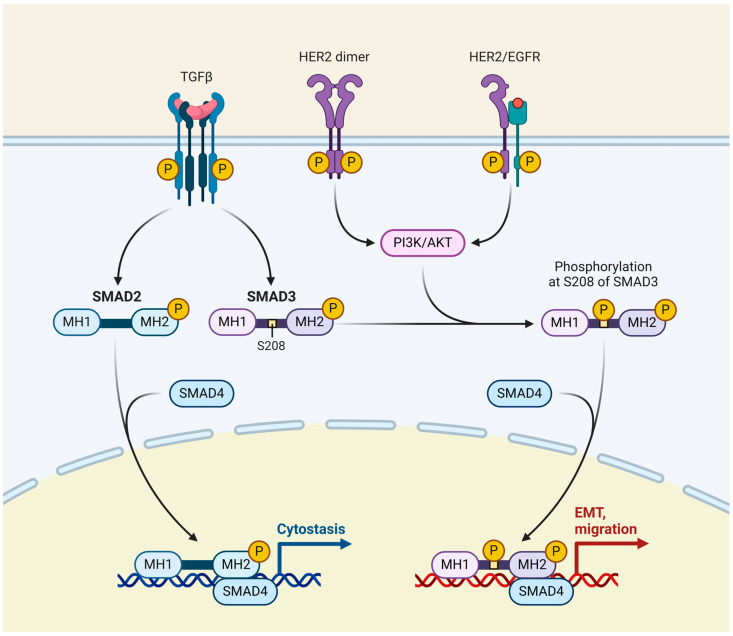
HER2/epidermal growth factor receptor (EGFR) signalling pathway in breast cancer. The HER2/EGFR signalling pathway in breast cancer involves the activation of HER2 and EGFR, which are tyrosine kinase receptors. When these receptors are overexpressed or mutated, they dimerise and activate downstream signalling cascades, including the PI3K/AKT and MAPK pathways. This leads to increased cell proliferation, survival, and metastasis. HER2-positive breast cancers are particularly aggressive but can be targeted with therapies like trastuzumab and pertuzumab, which block HER2 signalling.

In breast cancer, BsAbs have shown significant potential in preclinical and clinical studies [192]. For example, BsAbs targeting the HER2 can effectively direct immune cells to HER2-positive cancer cells, leading to their destruction. This is especially important for aggressive HER2-positive breast cancers (Figure 7), which often have poorer prognoses. Moreover, BsAbs that target immune checkpoints, such as PD-1 or PD-L1, can help overcome the immunosuppressive environment of tumours, enhancing immune-mediated tumour clearance [193]. The combination of BsAbs with conventional therapies, such as chemotherapy and immune checkpoint inhibitors (ICIs), has demonstrated synergistic effects, further improving their efficacy. Ongoing research aims to optimise the design, dosing, and combination strategies of BsAbs to maximise their therapeutic potential in breast cancer treatment [193].

Despite the promising potential of BsAbs in breast cancer therapy, several challenges remain. The complexity of BsAb design and production, potential immunogenicity, and the need for precise patient selection criteria are significant hurdles that need to be addressed. Moreover, managing the balance between efficacy and toxicity is crucial, as BsAbs can induce cytokine release syndrome and other immune-related adverse effects. BsAbs represent a novel and promising therapeutic strategy in breast cancer, offering the potential for more effective and targeted treatments. Ongoing research and clinical trials will be crucial in overcoming current challenges and fully realising the therapeutic potential of BsAbs. As the field advances, BsAbs may become a cornerstone in the treatment of breast cancer, providing new hope for patients with this challenging disease.

## 11. Discussion and Conclusions

Tumour dormancy is still an important but mysterious stage in the course of breast cancer. Though important, the precise processes controlling tumour cell dormancy are still poorly understood. Because dormant cancer cells may either completely stop growing or proliferate extremely slowly, they can be difficult to find and treat. Knowledge of the molecular mechanisms behind tumour dormancy may open the door to focused therapies meant to stop dormant cells from reactivating and spreading. One possible therapeutic approach is to reactivate dormant tumour cells so they become vulnerable to cytotoxic drugs; alternatively, methods to stop their reactivation may turn breast cancer into a controllable chronic illness. However, overcoming these obstacles will require closing major research gaps in breast cancer, such as finding hibernating cancer cells, deciphering molecular dormancy mechanisms, looking into the tumour microenvironment and metabolic adaptations, and creating new treatments and biomarkers for detection and monitoring. Furthermore, epigenetic changes mediated by G9a-enzymes become important factors in the recurrence of breast cancer, affecting important cellular functions connected to the cancer’s development, dormancy, and resistance to therapy. Extensive research aiming at comprehending the epigenetic processes mediated by G9a provide important information for developing novel treatment plans to fight breast cancer that returns. In controlling breast cancer recurrence, overcoming the changing obstacles related to G9a-targeted treatments has potential in regard to increasing therapy efficacy and improving patient outcomes. Through the filling in of these gaps and the use of new discoveries, scientists can further our knowledge of breast cancer and eventually enhance methods for its early detection, treatment, and long-term control.
